# Effect of Mental Fatigue on Postural Sway in Healthy Older Adults and Stroke Populations

**DOI:** 10.3390/brainsci10060388

**Published:** 2020-06-19

**Authors:** Gonzalo Varas-Diaz, Lakshmi Kannan, Tanvi Bhatt

**Affiliations:** 1 Department of Physical Therapy, University of Illinois at Chicago, Chicago, IL 60612, USA; gvaras2@uic.edu (G.V.-D.); lkanna2@uic.edu (L.K.); 2 College of Applied Health Sciences, University of Illinois at Chicago, Chicago, IL 60612, USA

**Keywords:** mental fatigue, postural sway, balance control

## Abstract

The aim of this study was to examine the effect of mental fatigue on postural sway under different sensory conditions in healthy older adults and in persons with chronic stroke (PwCS). Thirty healthy older adults (> 60 years old), randomly separated into experimental and control groups, as well as 15 PwCS participated in this study. Experimental groups were asked to stand on a force platform wearing seven inertial sensors while performing the Sensory Organization Test (SOT) under two cognitive conditions (single- and dual-task) before and after a mental fatigue task (stop-signal task for 60 min). The control group performed the same protocol before and after watching a movie for 60 min. Changes in subjective fatigue was assessed by the NASA Task Load Index and psychophysiological workload was assessed by heart rate variability (HRV). Postural sway was assessed by calculating the Jerk and root mean square (RMS) of center of mass (COM). Higher Jerk and RMS of COM (*p* < 0.05) were observed after the mental fatigue task in both healthy older adults and PwCS during SOT, which was not observed in the control group (*p* > 0.05). Additionally, postural sway increased in the three groups as the SOT conditions became more challenging. Our results indicate that mental fatigue, induced by sustained cognitive activity, can impair balance during SOT in older adult and stroke populations.

## 1. Introduction

Fatigue is one of the most common and disturbing symptoms in stroke and aging populations [1,2,3], with negative impacts on quality of life, self-esteem, and employability [4,5]. Several authors have described fatigue as a subjective multidimensional experience, with perceptual-motor, emotional, and cognitive components associated with an intense feeling of physical and/or mental overwork, even in the absence of special effort [1,2,6]. An important component of fatigue is mental fatigue, defined as a psychobiological state induced by sustained periods of demanding cognitive activity and characterized by feelings of tiredness which are common in everyday life [6]. Although some authors prefer to use the term cognitive fatigue to describe the psychobiological state associated with sustained cognitive activity, there is a consensus to use the term mental fatigue to include motivational and emotional aspects associated with task accomplishment [6].

Evidence indicates that older adults [2], people with cognitive impairments [7], and people with neurological disorders [3] often experience mental fatigue to a greater extent than young adults and their healthy counterparts that can impact the performance of daily activities [6]. Similarly, when these populations perform dual-task paradigms, in which postural control and cognitive tasks are performed simultaneously, they experience greater cognitive-motor interference demonstrated by performance deteriorations in either motor and/or cognitive tasks [8,9,10]. In this regard, studies have shown that both older adults and persons with stroke demonstrate a motor-related cognitive interference, in which they prioritize the motor tasks such as maintaining their limits of stability or gait speed controlling their postural sway while compromising performance on the cognitive tasks [9,10].

While dual-task paradigm studies give us an indication of how tasks that share cognitive and motor resources might be affected when neural resources are limited, as seen in aging or in neurological diseases, the cognitive load induced in these protocols is brief (lasting only several seconds or couple minutes), which could underestimate the effect of cognitive load on motor performance, explaining the predominant motor prioritization seen in older adults and in persons with stroke [9,10]. On the other hand, mental fatigue, caused by sustained overloading/overuse of cognitive functions, could also attenuate cognitive resource allocation for activities of daily living affecting their optimal functioning [1,6]. Thus, the relationship between actual induced mental fatigue and motor performance has elicited significant interest in the field and might be important to investigate [6]. Recent studies have reported that mental fatigue affects cognitive performance in older adults, such as difficulties in focusing their attention, increasing their reaction time, and increasing the number of errors during standardized cognitive tasks [11,12]. However, mental fatigue does not only affect cognitive task performance but also affects motor behavior. Thus, some studies have also shown that motor performance is decreased when mental fatigue is induced [8,13,14,15,16]. For example, Behrens et al. (2018) observed increased gait variability, assessed by the coefficient of variation of speed, stride length, and stance time after mental fatigue in older adults [17]. As increased gait variability has been described as a fall-risk predictor, the authors concluded that mental fatigue could be an intrinsic risk factor for accidental falls [17]. Additionally, it has been reported that mental fatigue led to an increased likelihood of slip initiation, poorer slip detection, and a more inefficient reactive recovery response to laboratory-induced slip-like perturbations in young adults [18], further suggesting that mental fatigue could be a risk factor for falls. However, while these studies involved challenging dynamic balance tasks, it remains to be determined whether mental fatigue could also interfere with more static postural tasks which are still essential for carrying out certain simple activities of daily living. In that context, although maintaining stable upright posture requires minimal attention, it has been observed that impaired cognitive functioning among older adults and stroke populations could cause balance instability and increase the risk of falls [19,20].

According to our knowledge, no study exists that has investigated the impact of mental fatigue, induced by sustained cognitive activity, on stance balance performance under different sensory conditions in older adults and in persons with chronic stroke (PwCS). In consideration to this, we assessed standing postural sway during the Sensory Organization Test (SOT), a balance test consisting of six conditions with altered sensory inputs (vision, proprioception, and vestibular), before and after a randomly assigned mental fatigue and control interventions in healthy older adults and in PwCS. Thus, the primary aim of this study was to examine the effect of mental fatigue, induced by sustained cognitive activity, on postural sway during the SOT under single- and dual-task conditions. Postural sway was assessed via the root mean square (RMS) and Jerk of center of mass (COM). We hypothesized that mental fatigue impairs balance control and increases the sway of the COM control during SOT especially while performing a concurrent attention-demanding cognitive task (dual-task condition) and as the SOT condition becomes more challenging among both older adults and PwCS. The secondary aim of this study was to compare the effect of mental fatigue on postural sway during the SOT between older adults and PwCS. For this, we hypothesized that the postural sway in PwCS would be significantly more affected by mental fatigue compared to their age-similar healthy older adults.

## 2. Materials and Methods

### 2.1. Participants

Thirty healthy older adults (> 60 years old) (19 females and 11 males, age (67.6 ± 7.1) were assigned either to an intervention group or to a control group (each group was composed of 15 participants). Additionally, fifteen people with self-reported hemiparetic chronic stroke (>6 months) (nine females and six males, age 63.8 ± 8.6) formed a second intervention group. All participants provided written informed consent and this study was approved by the Institutional Review Board in the University of Illinois at Chicago (Protocol ID: 2018-1091, approval date: October 17, 2018)

Healthy older adults were included if they passed a cognitive test (> 26/30 on Montreal Cognitive Assessment Scale) and a mobility test (Timed Up and Go (TUG) SCORE < 13.5) to ensure that these individuals were independent ambulators without balance or gait impairments. Individuals were excluded if they self-reported any neurological, musculoskeletal, or other systemic disorders that would affect the subject’s postural control. On the other hand, persons with chronic stroke (PwCS) (> 6 months) were included if they were able to ambulate independently with or without an assistive device. Participants with cognitive deficits (score of < 26/30 on Montreal Cognitive Assessment Scale), speech deficits (aphasia score of > 71/100 on Mississippi Aphasia Screening Test), or presence of any other self-reported neurological, musculoskeletal, or cardiovascular conditions were excluded from the study. Baseline functional status assessments, including the number of years since stroke, severity of impairment (Chedoke–McMaster Stroke Assessment), and balance testing (Berg Balance Scale, and TUG) were performed. Demographic details and baseline clinical assessments are presented in Table 1.

### 2.2. Experimental Protocol

**Figure 1:**
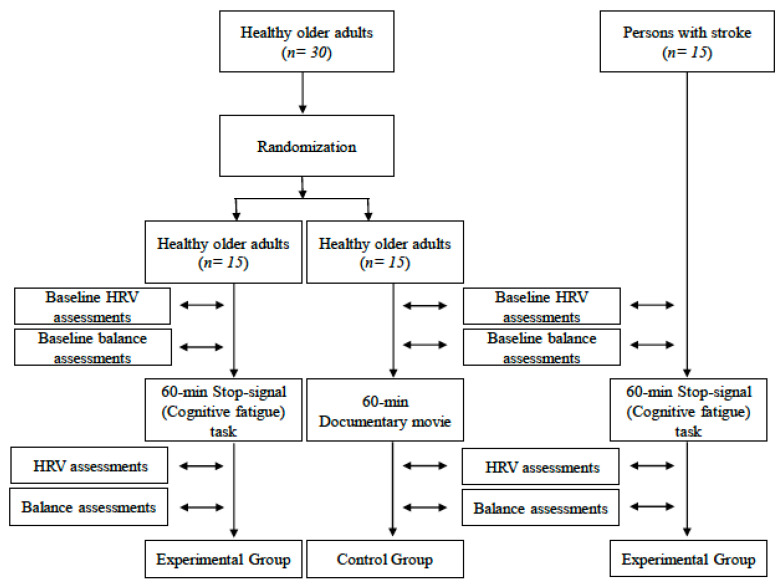
Experimental protocol scheme. HRV, heart rate variability.

This study employed a randomized design in which thirty healthy older adults were randomized to either an experimental or a control group. Randomization was according to a random number (either 0 or 1) generated by Microsoft Excel ‘‘RAND” function (Microsoft Office 2016, v 16.0, Chicago, IL, USA). Additionally, PwCS formed a second experimental group. During the experiment, 15 healthy older adults and 15 PwCS (experimental groups) were asked to stand on a platform wearing seven MTX Xsens inertial sensors while performing the six sensory conditions included in the Sensory Organization Test (SOT) of the Balance Master under two cognitive conditions (no cognitive task and serial subtractions (SS) task) before and after a cognitive fatigue task (stop-signal task for 60 min) programed by DirecRT_tm_ Software v 2016 (Empirisoft Corporation, New York, NY, USA) (http://www.empirisoft.com/DirectRT.aspx). Also, 15 healthy older adults (control group) performed the same protocol before and after watching a documentary movie for 60 min. Before and after each intervention, heart rate variability (HRV), as a physiological indicator of mental fatigue [21], and postural sway, assessed by Jerk and RMS of COM, under single- and dual-task conditions during SOT were recorded (Figure 1). After each intervention, subjective fatigue, assessed by the NASA Task Load Index, was conducted.

### 2.3. Cognitive Fatigue and Control Intervention Tasks

**Figure 2:**
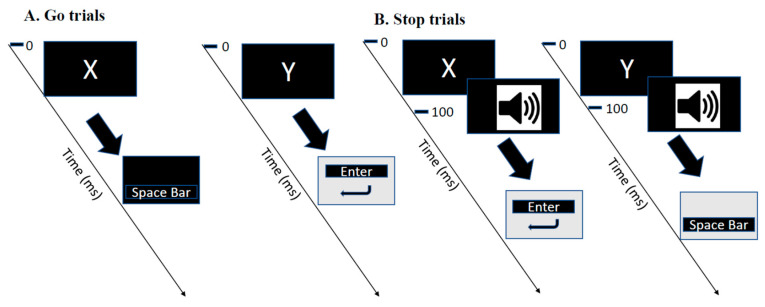
Experimental protocol for cognitive fatigue task. (**A**). Participants were asked to respond to a letter X and Y pressing the “Space bar button” and “Enter button,” respectively. (**B**). On a subset of trials, this was followed by an auditory stop signal, which signaled participants to reverse their response. ms, milliseconds.

During the cognitive fatigue intervention, participants had to perform a stop-signal task [22] for 60 min on a laptop computer. This task is a commonly used laboratory measure of inhibitory control that consists of presenting concurrent go and stop tasks. Participants had to press the “Space” or the “Enter” button as quickly and accurately as possible in response to the visual presentation of the letter X or Y, respectively, using their dominant hand. The stop signal consisted of a delayed tone (100 ms after the initial stimuli) presented by headphones and when it occurred, it signaled the participants to now reverse their response and do the opposite—that is, to press the “Space bar” button in response to the letter Y and the “Enter” button in response to the letter X (Figure 2). The percentage of correct answers was computed to monitor performance during the mental fatiguing task [23]. The average of this parameter was calculated for five blocks during the stop-signal task. The control intervention consisted of watching the documentary “Earth” for 60 min on the same computer used for the mental fatigue task.

### 2.4. Postural Sway Assessment

The SOT consists of six conditions designed to separate the sensory effects of vision, proprioception, and vestibular input during standing balance [24]. The protocol consists of the following conditions: the eyes open firm surface (EOF) condition, in which participants were asked to maintain standing balance on a solid support surface with eyes open; the eyes closed firm surface (ECF) condition, in which participants were asked to maintain standing balance on a solid support surface with eyes closed; the eyes open with sway referenced vision (EOSV) condition, in which participants had to maintain the standing position on solid support surface, sway referenced surround with eyes open; the eyes open sway referenced surface (EOSS) condition, in which participants had to maintain the standing balance on a sway referenced support surface with eyes open; the eyes closed sway references surface (ECSS) condition, in which participants had to maintain standing balance on a sway referenced support surface with eyes closed; and the eyes open, sway referenced surface and vision (EOSSV) condition, in which participants had to maintain standing balance on a sway referenced support surface, sway referenced surround with eyes open [24,25]. It has been reported that balance control may be more sensitive to cognitive manipulations, such as cognitive load or cognitive fatigue, during challenging sensory conditions [10]. Therefore, we decided to analyze the last three conditions of the SOT, including the EOSS, ECSS, and EOSSV conditions.

In order to calculate the postural sway under different sensory conditions included in SOT, participants wore MTX Xsens sensors (49A33G15, XSens, Enschede, The Netherlands) with 3D accelerometers (± 1.7 g range), and 3D gyroscopes, (± 300°/s range) mounted on the posterior low back at the level of L5 (near the body center of mass), and at both lower extremities. The sensing axes were oriented along the anatomical anteroposterior (AP), mediolateral (ML), and vertical directions.

For each trial performed during the SOT, two variables were calculated from the resultant 2D acceleration (Acc) measured at the L5 level with respect to the base of support: (1) root mean square acceleration (RMS), which quantifies the magnitude of Acc traces [25]; and (2) the resultant Jerk of COM value, an indicator of the smoothness of postural sway, which was computed as follows [25,26]:

$$\mathrm{jerk}=\frac{1}{2\;}\int_{0}^{T}\left({\left(\frac{\mathrm{dAccAP}}{\mathrm{dt}}\right)}^{2}+{\left(\frac{\mathrm{dAccML}}{\mathrm{dt}}\right)}^{2}\right)$$

where AccAP and AccML are the acceleration components measured in the AP and ML directions, respectively. As a function of the time derivative of the acceleration, Jerk can be seen as a measure of the ability to control and/or to decelerate motion and as a measure of dynamic stability.

### 2.5. Dual-Task Protocol

In addition to the single-task balance test, outcome measures during postural sway assessment were also recorded while performing a concurrent attention-demanding cognitive task (without explicit instructions regarding prioritization). The task consisted of serial numerical subtractions, starting from a randomly selected number between 90 and 200. The results of this arithmetic task had to be recited verbally by the participants, and the cognitive interference task performance was calculated by subtracting the number of errors from the total number of subtractions. The higher the value, the better the performance [27].

### 2.6. Psychophysiological Workload

Mental fatigue is associated with the alteration of activity in multiple peripheral homeostatic pathways in response to acute stressors in older adults [6]. Additionally, mental fatigue and heart rate variability (HRV), particularly the parasympathetic markers derived from HRV analysis, are related to selective regions of the prefrontal cortex, basal ganglia, insula, and anterior cingulate cortex. These areas build a functional circuit called the fronto-basal ganglia circuitry which deeply involves executive functions [28]. In this regard, the psychophysiological workload produced by the 60 min mental fatigue and control designated task was analyzed using HRV [21]. Heartbeat intervals were continuously recorded for 10 min in the supine position, before and immediately after the intervention (mental fatigue or control intervention). These intervals were recorded using a heart rate monitor (Polar RS800CX, Polar Electro Oy, Kempele, Finland), with a sampling rate of 1000 Hz, which wirelessly receives heart rate data from a chest strap (two-lead) worn by the participants [20,28].

The raw data were extracted from a text file stored in the polar acquisition software POLAR PRO trainer 5 (Polar Electro^TM^, OY, Kempele, Finland) and imported into HRV analysis Kubios software (version 4.0, 2012, Biosignal Analysis and Medical Imaging Group, University of Eastern Finland Kuopio, Finland, MATLAB) [29].

Linear statistical measures were performed in the time and frequency domains [30]. The frequency domain of HRV methods uses the power spectral density that measures how power distributes as a function of frequency. Spectral analysis of HRV signal, recorded from beat to beat variations in heart rate spectral components, which in turn differentially reflect autonomic mediators of cardiovascular variability including high-frequency (HF) and low-frequency (LF), were expressed in normalized units (nu) [29,30]. All the frequencies were obtained using the Fast Fourier Transformation.

In the time domain analysis, the root mean square of differences between adjacent normal RR intervals (RMSSD) in a time interval was analyzed [30].

### 2.7. Statistical Analysis

Data were screened for normal distribution using the Shapiro–Wilk test. A two by two analysis of variance (ANOVA) was conducted to test the effect of the intervention on the psychophysiological workload, assessed by HRV variables. A one-way ANOVA was conducted to test the subjective fatigue after the intervention task, assessed by the NASA Task Load Index. Two separate multivariate analyses of variance (MANOVA) were conducted with time (pre- vs. post-mental fatigue task), group (older adults, stroke group, and control group), task (single vs. dual-task), and SOT conditions (EOSS, ECSS, EOSSV) as independent variables for each of the dependent postural control measures–jerk of COM and RMS of COM. The significant main effects and interactions were resolved using planned repeated-measures ANOVA and were followed up with posthoc tests. To verify the magnitude of the changes after the intervention, the effect size (ES) was calculated based on Cohen’s *d*. Effect size is classified as small (*d* = 0.0–0.20), medium (*d* = 0.30–0.50), and large (*d* = 0.50–0.80). The level of statistical significance was set at *p* ≤ 0.05. Data were analyzed using the SPSS package 22.0 (SPSS Inc., Chicago, IL, USA).

## 3. Results

There was no demographic (age, weight, height) differences between participants from the intervention groups and control group. Additionally, the scores of the MOCA test (28.3 ± 2.6) indicated that participants from all the groups were cognitively healthy (Table 1). Regarding baseline cardiovascular parameters, baseline heart rate assessment, and mean of R-R intervals (time in seconds between heartbeats) were not significantly different between the three groups (*p* > 0.05) (Table 2).

### 3.1. Psychophysiological Workload and Subjective Fatigue

The two-way ANOVA for RMSSD values displayed a significant main effect of time (*F* (*1*,*42*) = 10.873, *p* < 0.01) and group by time interaction (*F* (*2*,*42*) = 3.059, *p* = 0.048). However, no group effect (*F* (*2*,*42*) = 0.656, *p =* 0.524) was observed. Similarly, for HRV HF power, we observed a significant main effect of time (*F* (*1*,*42*) = 24.001, *p* < 0.01), but there was no group by time interaction (*F* (*2*,*42*) *=* 2.053, *p* = 0.141) or group effect (*F* (*2*,*42*) = 0.336, *p* = 0.701) observed. Post-hoc analyses revealed that RMSSD values and HRV HF power were lower after cognitive fatigue task compared to the baseline assessment (*p* < 0.05) for the experimental older adult group and for the stroke group (both experimental groups). For the control group, no differences in RMSSD values and HRV HF power were observed between pre- and post-control tasks (*p* > 0.05) (Table 2).

The one-way ANOVA for subjective fatigue revealed a significant main effect of group (*F* (*2*,*42*) = 122.41, *p* < 0.01) for the NASA Task Load Index performed after the intervention, in which higher scores were observed in the stroke group compared to the experimental older adult group and control group (*p* ≤ 0.05), and in the experimental older adults group compared to the control group (*p* ≤ 0.05).

### 3.2. Balance Assessments

The MANOVAs revealed that the cognitive fatigue task differentially affected postural sway (Jerk of COM and RMS) among the three groups (older adults, stroke, and control group) as indicated by a significant group by time effect. Additionally, these groups performed significantly different from each other during the SOT conditions (EOSS, ECSS, EOSSV) as indicated by a significant group by the SOT condition effect. However, the type of task (single- and dual-task) did not have significant changes in balance control measures (jerk of COM and RMS) post-intervention. The details of these results are presented in Table 3A.

Considering there was a minimal interaction effect of task (single- and dual-task) on other independent variables, the significant MANOVA’s effects were resolved by performing separate repeated-measures ANOVA under single- and dual-task performances to determine the differences in pre- compared to post-mental fatigue between groups for each SOT condition (Table 3 and Table 4).

#### 3.2.1. Jerk of COM during Single-Task Assessments

**Figure 3:**
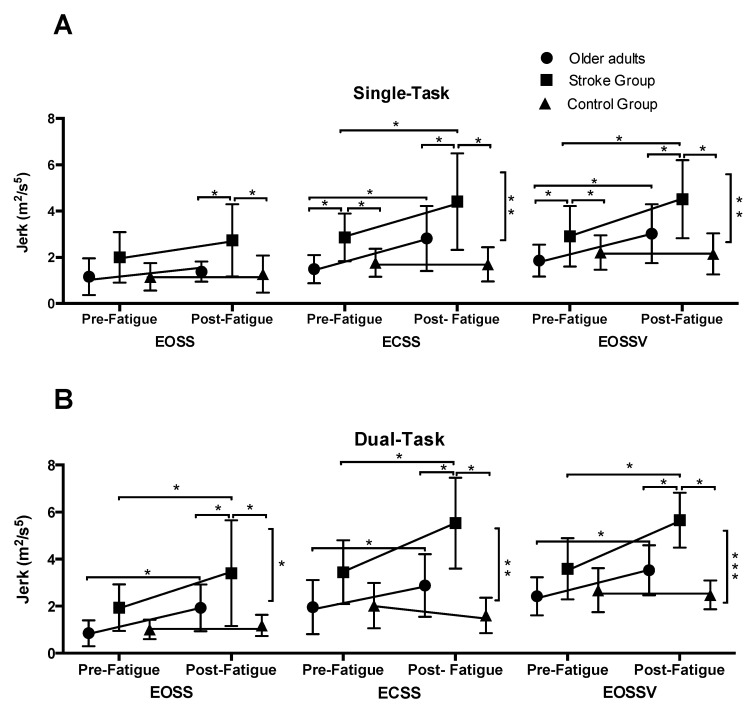
Shows the Jerk of COM values in each SOT condition for the older adults, stroke, and control groups recorded before and after the mental fatigue and control intervention, respectively, during the (**A**). Single-task protocol. (**B**). Dual-task protocol. Vertical bars represent the time by group effect. * *p* ≤ 0.05 ** *p* ≤ 0.01 *** *p* ≤ 0.001. Data are presented as the mean ± standard deviation.

Under single-task conditions, repeated-measures ANOVA revealed a significant main effect of time, group, and SOT conditions, and time by group effect (Table 3B). Overall, there was an increase in a jerk as the SOT condition increased in difficulty (EOSSV > ECSS > EOSS). Resolving the time by group interaction for each condition revealed that for all the SOT conditions there was a significant main effect of time and group. Additionally, a time by group interaction was observed for ECSS and EOSSV conditions. The details are provided in Table 4A. Posthoc analysis revealed that post-intervention, both experimental groups increased their Jerk values during ECSS and EOSSV conditions (*p* ≤ 0.05). On the other hand, no pre- to post-changes were observed for the control group in any of the SOT conditions (*p* > 0.05) (Figure 3A). Further, compared to the experimental older adult group and the control group, the stroke group exhibited increased Jerk values (*p* ≤ 0.01) during all the SOT conditions included in the study protocol (Figure 3A).

#### 3.2.2. Jerk of COM during Dual-Task Assessments

Under dual-task conditions, repeated-measures ANOVA revealed a significant main effect of time, group, and SOT conditions, and time by group effect (Table 3B). Overall, Jerk values were higher as the SOT condition increased in difficulty (EOSSV >ECSS > EOSS). Resolving the time by group interaction for each SOT condition, there was a significant main effect of time and group as well as a time by group interaction. The details are provided in Table 4B. Post-hoc analysis revealed that post-intervention, both experimental groups increased their jerk values during EOSS, ECSS, and EOSSV conditions (*p* < 0.05). On the other hand, no pre- to post-changes were observed for the control group in any of the SOT conditions (*p* > 0.05) (Figure 3B). Further, compared to the experimental older adult group and the control group, the stroke group exhibited increased jerk values (*p* ≤ 0.001) during all the SOT conditions included in the study protocol (Figure 3B).

#### 3.2.3. Root Mean Square during Single-Task Assessments

**Figure 4:**
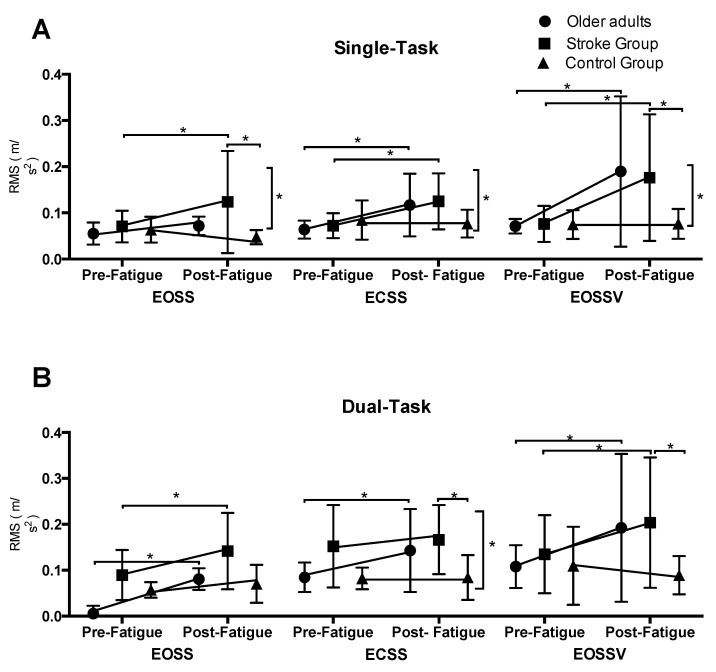
Shows the root mean square (RMS) of COM values in each SOT condition for the older adults, stroke, and control groups recorded before and after the mental fatigue and control intervention, respectively, during the (**A**). Single-task protocol; (**B**). Dual-task protocol. * *p* < 0.05. Vertical bars represent the time by group effect. Data are presented as the mean ± standard deviation.

Under single-task conditions, repeated-measures ANOVA revealed a significant main effect of time, group, and SOT conditions, time by group interaction, and a time by SOT condition interaction (Table 3B). Overall, there was an increase in RMS as the SOT condition increased in difficulty (EOSSV>ECSS>EOSS). Resolving the time by group interaction for each SOT condition, there was a significant main effect of group and a time by group interaction for the EOSS condition. For ECSS and EOSSV conditions, there was a significant main effect of time and time × group interaction (Table 4A). Post-hoc analysis revealed that post-intervention, both experimental groups significantly increased their RMS compared to the baseline assessments (*p* < 0.05). No pre- to post-changes were observed for the control group in any of the SOT conditions (*p* > 0.05) (Figure 4A). Further, compared to the control group, the stroke group exhibited increased RMS values during EOSS and EOSSV (*p* ≤ 0.05). No differences were observed between both experimental groups in any of the SOT conditions (Figure 4A).

#### 3.2.4. Root Mean Square during Dual-Task Assessments

Under dual-task conditions, repeated-measures ANOVA revealed a significant main effect of time, group, and SOT conditions, and time by group effect (Table 3B). Overall, there was an increase in RMS as the SOT condition increased in difficulty (EOSSV > ECSS > EOSS). Resolving the time by group interaction for each condition, there was a significant main effect of time and group for all SOT conditions. In addition to this, a time by group interaction was observed for the ECSS condition (Table 4B). Posthoc analysis revealed that post-intervention, both experimental groups increased their RMS values during the ECSS condition (*p* < 0.05). No pre- to post-changes were observed for the control group in the ECSS SOT condition (*p* > 0.05) (Figure 4B). Further, compared to the control group, the stroke group exhibited an increased RMS values during ECSS and EOSSV conditions (*p* ≤ 0.05). No differences were observed between both experimental groups in any of the SOT conditions (Figure 4B).

### 3.3. Performance during Cognitive Fatigue Intervention

No differences were observed in the percentage of correct answers (*p* > 0.05) between both experimental groups (older adults and stroke) during the stop-signal task (Table 5).

### 3.4. Performance during Dual-Task Protocol

A significant main effect of time (*F* (*1*,*42*) = 62.232, *p* < 0.01) and interaction effects of time and group (*F* (*2*,*42*) = 8.233, *p* < 0.01) were observed for the serial subtraction task during the dual-task protocol. Post-hoc analysis revealed that the cognitive performance decreased significantly in both the experimental older adult group (*p* < 0.01) and the stroke group (*p* < 0.01) after performing the cognitive fatigue task. No differences in cognitive performance during the dual-task protocol between pre- and post-intervention tasks were observed for the control group (Table 5).

## 4. Discussion

The aim of the present study was to investigate the effect of mental fatigue, induced by sustained cognitive activity, on postural sway while performing the SOT under single- and dual-task conditions in healthy older adults and persons with chronic stroke (PwCS). Consistent with our hypothesis, postural sway, assessed by Jerk of COM and RMS in the both AP and ML directions, increased after a 60 min cognitive fatigue task (stop-signal task) in both healthy older adults and PwCS during SOT, with more challenging SOT conditions demonstrating greater postural sway. However, contrary to our hypothesis, the cognitive fatigue tasks did not differentially affect postural sway under single-task versus dual-task conditions. These findings suggest that mental fatigue could be seen as a potential intrinsic risk factor for balance disorders in healthy older adults and PwCS.

### 4.1. Effect of Mental Fatigue on Psychophysiological Workload and Subjective Fatigue

The results indicated that mental fatigue was induced successfully in our participants as demonstrated by changes in self-reported state of fatigue and wakefulness, assessed by the NASA Task Load Index. Further, this was also shown by a decrease in parasympathetic activity after the cognitive fatigue task compared to the baseline assessment, measured by time and frequency domains HRV parameters (Table 2), which is in line with previous studies that have used a similar computer-controlled task to provoke mental fatigue [17,31]. In this regard, time domain and frequency domain HRV analyses have been widely used to investigate the cardiovascular consequences of mental work and cognitive load [21,32]. These show that increased levels of mental fatigue lead to a decrease in the time domain measures, as well as a decrease in the LF and HF powers, while the LF/HF ratio increases [21,32]. This is postulated to occur due to a predominant decrease in the parasympathetic activity and/or a predominant increase in the sympathetic activity after cognitive workload [21,30,32] influenced by cortical changes in brain areas related to executive functions [28].

Brain areas related to executive control, including prefrontal cortex (PFC) and anterior cingulate cortex (ACC), have been shown to play an important role in autonomous nervous system regulation. These regions inhibit the sympathoexcitatory nucleus located in subcortical areas and contribute to the balance between parasympathetic and sympathetic outflows [28,32,33]. Under stress and workload conditions, it has been shown that PFC and ACC decrease the neural activity by reducing parasympathetic and increasing sympathetic outflow, which is interpreted as a state of autonomic hypervigilance and sympathoexcitatory activity triggered by subcortical circuits that are normally under inhibitory control of the PFC [28,32,33].

### 4.2. Effect of Group and Sensory Conditions on Postural Sway

For older adults, baseline results (pre-cognitive fatigue task) revealed an increase in Jerk and RMS of COM as the level of difficulty of the postural task increased (from EOSS to EOSSV SOT conditions). This result is consistent with previous findings which have reported that by increasing the level of postural difficulty, the amount of COP variability and postural sway increases [34,35]. It is known that the body’s postural control system possesses the ability to “inhibit” poor sensory cues and “promote” reliable and consistent cues, known as sensory reweighting, which is indicated to become inefficient with aging [36,37]. In our study, as the sensory and kinesthetic information was progressively reduced or made inaccurate progressing from EOSS to EOSSV conditions, the participant was required to reweight sensory input. Inefficient sensory reweighting could have caused the individuals to experience prolonged instability, thereby exhibiting increased postural sway. Moreover, from a physiological perspective, disturbances in visual and somatosensory information are known to increase the difficulty of postural control, resulting in decreased postural stability [34]. This is evidenced by the increased amount of COP variability (increased SD of velocity) and increased postural sway, previously demonstrated in older adults [24,34,35]. Similarly, our study showed that for ECSS and EOSSV SOT conditions, in which more than one sensory input was altered (ECSS—visual and somatosensory information were obscured; EOSSV—somatosensory and visual inputs were inaccurate), postural sway significantly increased in all groups. In this regard, maintaining balance control when sensory input is being altered challenges the postural control system to process adaptive sensory reweighting [37]. For example, sway-referencing the surface under a subject who has their eyes closed (ECSS) or looking at a sway-reference visual surround (EOSSV) requires a person to depend more upon vestibular inputs to control balance, which can be impacted by aging [37]. Thus, our results are in line with other studies, indicating a decreased ability of older adults to process adaptive sensory reweighting [37].

Similar to the healthy older adult group, PwCS displayed decreased postural control as the sensory conditions got more challenging (Figure 3 and Figure 4). However, our results demonstrated that, at baseline, Jerk and RMS of COM were significantly greater in PwCS compared to the healthy older adult groups for most of the SOT conditions. This could be explained because PwCS exhibit several sensory and motor impairments which cause asymmetrical posture and weight-bearing as well as decreased muscle strength—all of which are essential factors to maintain balance control [34,38]. Such impairments may further affect the ability to process adaptive sensory reweighting thereby increasing postural sway compared to healthy older adults. Along this line, PwCS have shown two-folded increases in postural sway compared to healthy older adults [38]. The findings of our study are similar to previous studies in that the dependency on visual and somatosensory information for maintaining postural control is significantly higher in PwCS relative to healthy older adults [37]. This could suggest that the internal representation of the postural body scheme is affected due to altered mechanisms of sensorimotor integration in supraspinal centers in PwCS [39]. Further, the stroke-induced brain insult might affect cortical proprioceptive processing centers, thus further compromising postural sway, especially when more than one sensory information source is absent [37,38].

### 4.3. Effect of Mental Fatigue on Postural Sway

As hypothesized, our results allow us to infer that mental fatigue can affect posture control in older adults and PwCS, and this effect is magnified under challenging sensory conditions (i.e., when more than one sensory input is disturbed). The effect of mental fatigue was not observed on Jerk of COM values during the EOSS condition of SOT (Figure 3A), in which only one sensory input was disturbed (proprioception). However, in the ECSS condition, in which visual input is canceled and proprioceptive inputs were altered, and in the EOSSV condition, in which visual and proprioceptive inputs are manipulated and put in conflict, the effect of mental fatigue on postural sway was clearly observed in both older adults and in persons with stroke (Figure 3 and Figure 4). This suggests that the control of postural sway may be more sensitive to cognitive manipulations during challenging sensory conditions compared to more stable postural tasks. In line with our findings, Mehdizadeh et al. (2015) showed that persons with stroke and healthy older adults present greater velocity of the center of pressure (COP) during a difficult compared to a simple memory task or no cognitive task while standing on foam with eyes closed (visual information canceled and somatosensory information altered) [10]. This increase in COP velocity was not observed while the same participants performed similar cognitive tasks on a firm surface with eyes open or closed (no sensory conflict), thus confirming that balance control may be more sensitive to cognitive manipulations during more demanding sensory conditions [10].

It has been postulated that maintaining balance control requires a given amount of attentional resources which depend on the complexity of the postural task: the more challenging the postural task, the greater the required attentional resources [40,41]. It has also been shown that mental fatigue decreases the activity of PFC and ACC [33]—two of the most important brain areas involved in executive functioning—which are essential for attentional control processes during gait and balance tasks [42]. Thus, impaired executive functioning could result in poor self-awareness of physical limitations and further lead to inappropriate evaluation of environmental variables, affecting subsequent motor output [43]. The induction of a mental fatigue state, experienced by both experimental groups, could have an impact on the executive functioning and allocation of attentional resources required to perform the postural stability task especially under challenging environmental conditions. This could explain the differences in motor and cognitive performance observed between pre- and post-assessments in both of the intervention groups. Another plausible reason for the deterioration in postural sway after mental fatigue could be attributed to the possible structural alterations of the brain, known to occur with aging or pathology, such as a decreasing of neural connectivity in prefrontal areas and brain dysmorphology including ventricular enlargement and white matter hyperintensities [36,43]. Such structural changes could possibly decrease attentional capacity and cognitive processing ability while significantly increasing cognitive motor interference, especially during a state of mental fatigue. Thus, reduced attentional resources and availability of executive networks under mental fatigue combined with alterations of brain morphology (as in aging and stroke) could lead to a significant reduction in available cognitive resources that might be required for maintaining dynamic postural control, especially under challenging sensory conditions [40].

### 4.4. Effect of Mental Fatigue on Dual-Task Performance

One part of our hypothesis stated that the impact of mental fatigue on postural sway in healthy older adults and PwCS would be higher while performing a concurrent attention-demanding cognitive interference task (dual-task protocol). Although postural sway, measured by Jerk and RMS of COM, was higher during dual- compared to single-task for the three groups during the baseline assessment, Jerk and RMS values were not significantly different between single- and dual-task for any of the three groups after inducing mental fatigue (Table 3A,B). As was described before, postural sway increased significantly after the cognitive fatigue task in both experimental groups. This deterioration in postural sway was similar for single- and dual-task tests which allow us to infer that the effect of the induced mental fatigue was strong enough to similarly affect simple and complex balance tasks. However, another plausible explanation for this result could be accounted by the difficulty level of the dual-task paradigm implemented in this study. Previous studies have demonstrated that motor and cognitive performances are more affected during complex than simple dual-task situations [44]. As all the participants in this study were not cognitively impaired, it is possible that the dual-task protocol implemented was too simple to surface differences after the cognitive fatigue task in our participants. Finally, another possible explanation of these results is that participants could have prioritized motor instead of cognitive performance during the dual-task balance assessment after the cognitive fatigue intervention. In this regard, both the older adult and stroke groups showed a lower performance after the cognitive fatigue intervention in the serial subtraction task during the dual-task testing compared to the baseline assessment. This allows us to infer that while participants were experiencing a mental fatigue state, they prioritized the motor instead the cognitive performance during the dual-task testing.

The following limitations should be considered when interpreting the results of the current study. First, although the effect of mental fatigue on balance in older adults and persons with stroke was demonstrated, the sample size in each group was relatively small and a large number of comparisons were performed. Second, the control group included only older adult participants. Our study did not have an explicit control group to directly compare the effect of the cognitive fatigue task versus a passive observation task in PwCS. Future studies could include a specific control group for stroke populations to confirm the results observed in the current protocol. Finally, no specific psychological assessment was conducted. Due the relationship between psychological disorders such as depression and fatigue, future studies should address this limitation.

## 5. Conclusions

In conclusion, our results indicate that mental fatigue, induced by sustained cognitive activity, can impair postural sway measured during standing under varying sensory conditions performed with and without an additional cognitive task in healthy older adults and PwCS. These results allow us to infer that the susceptibility to mental fatigue could be seen as a new intrinsic risk factor for balance disorders and/or falls in older people and PwCS. Moreover, the potential influence of mental fatigue on balance should be considered when postural control analyses are performed in a scientific or clinical context. Future studies should investigate the underlying neural mechanisms of mental fatigue in populations with a high risk of falling. In addition, future studies should focus on the dose–response relationship between the extent of mental fatigue and the increase in balance disorders and/or risk of falls in older adults and in persons with neurological diseases.

## Figures and Tables

**Table 1:** Demographic trait and baseline clinical assessments.

Variables	Older Adult Group	Stroke Group	Control Group
Age (years)	66.1 ± 6.02	62.6 ± 5.2	65.6 ± 6.2
Weight (kg)	77.4 ± 12.06	75.6 ± 5.2	78.9 ± 4.4
Hight (cm)	173.67 ± 6	175.1 ± 4.7	178.3 ± 8.1
MOCA test Impairment level	27.4 ± 2.2	27.1 ± 1.8	28.1 ± 2.5
CMSA (leg)	-	5.18 ± 2.01	-
CMSA (foot)	-	4.6 ± 2.1	-
BBS	54.2 ± 1	47.4 ± 6 *	54.2 ± 1.3
TUG (s)	7.3 ± 2.8	13.7 ± 5.3 *	7.1 ± 1.3
FSS	21.2	22.4	21.5

Demographic and baseline clinical measures for study participants. Data are presented as the mean ± standard deviation. MOCA, Montreal Cognitive Assessment Scale; CMSA, Chedoke–McMaster Stroke Assessment; BBS, Berg Balance Scale; TUG, Timed Up and Go; kg, kilograms; cm, centimeters; s, seconds; FSS, Fatigue Severity Scale * *p* < 0.05.

**Table 2:** Psychophysiological workload parameters.

	Older Adult Group		Stroke Group		Control Group	
Cardiovascular Variables	Baseline	Post-Intervention Task	Baseline	Post-Intervention Task	Baseline	Post-Intervention Task
Mean of heart rate (beat/min)	64.6 ± 1.7	63.4 ± 3.5	66.3 ± 1.1	67.1 ± 3.1	62.8 ± 1.8	63.6 ± 4.7
Mean of R-R intervals	0.857 ± 0.03	0.840 ± 0.08	0.847 ± 0.07	0.820 ± 0.03	0.817 ± 0.02	0.822 ± 0.06
RMSSD (ms)	28.57 ± 7.3	19.51 ± 2.87 *	27.1 ± 16.98	22.22 ± 9.77 *	23.42 ± 9.38	23.62 ± 9.06
HRV HF power (n.u.)	26.04 ± 8.69	21.7 ± 12.8	27.49 ± 20.7	18.9 ± 16.45 *	21.38 ± 9.7	20.33 ± 6.20
**NASA-TLX**	-	-	-	-	-	-
Mental demand	-	68.4 ± 22.7	-	70.9 ± 31.9	-	13.4 ± 24.6
Physical demand	-	19.1 ± 11.5	-	27.5 ± 9.3	-	3.4 ± 6.7
Temporal demand	-	46.7 ± 38.1	-	55.8 ± 48.2	-	5.1 ± 13.6
Performance	-	59.8 ± 31.1	-	67.4 ± 33.8	-	91.4 ± 16.4
Effort	-	77.4 ± 23.6	-	81.3 ± 31.3	-	17.6 ± 15.5
Frustration level	-	39.6 ± 37.6	-	31.3 ± 35.6	-	4.7 ± 4.8
Global score	-	51.8 ± 22.6 **	-	55.7 ± 16.4 **	-	22.6 ± 12.8

Cardiovascular parameters pre- and post-intervention task, and the NASA Task Load Index dimension scores post-intervention task. * *p* ≤ 0.05 ** *p* ≤ 0.01. Data are presented as the mean ± standard deviation. RMSSD: root mean square of differences between adjacent normal RR intervals; HRV: heart rate variability; HF: high frequency; ms: milliseconds; n.u.: normalized units; TLX: Task Load Index.

**Table 3:** Overall results for Jerk and RMS of COM and ANOVA results for Jerk and RMS during single- and dual-task conditions.

**A**					
**Main effects and Interaction**	**Df (df1,df2)**	**Jerk of COM** ***F* Value**		**RMS** ***F* Value**	
Group effect	2,504	132.104 ***		23.74 ***	
Time effect	1,504	71.175 ***		37.496 ***	
Cognitive task effect (ST, DT)	1,504	16.021 ***		13.749 ***	
SOT condition effect	2,504	74.751 ***		20.782 ***	
Group × time effect	2,504	25.489 ***		11.811 ***	
Group × cognitive task (ST, DT)	2,504	2.533		2.249	
Group × SOT condition effect	2,504	2.485 *		1.833	
Time × cognitive task (ST, DT)	1,504	1.277		0.453	
Time × SOT condition	2,504	0.932		3.20 **	
Cognitive task × SOT condition	2,504	2.644		1.045	
Group × time × cognitive task (ST, DT)	2,504	0.914		0.477	
Group × time × SOT condition	4,504	1.273		1.872	
Group × cognitive task × SOT condition	4,504	0.157		0.498	
Time × cognitive task × SOT condition	2,504	0.801		0.966	
Group × time × cognitive task × SOT condition	4,504	0.305		0.129	
**B**					
	**Df (df1,df2)**	**Single-Task Condition**		**Dual-Task Condition**	
		**Jerk of COM** ***F* Values**	**RMS** ***F* Values**	**Jerk of COM** ***F* Values**	**RMS** ***F* Values**
Group effect	2,126	40.641 ***	6.627 **	65.021 ***	14.568 **
Time effect	1,126	37.733 ***	30.481 ***	59.493 ***	15.760 **
SOT condition	2,126	20.618 ***	7.092 ***	39.895 ***	11.168 **
Group × time	2,126	12.348 ***	10.607 ***	22.970 ***	4.511 *
Group × SOT condition	4,126	0.724	1.442	1.343	0.714
Time × SOT condition	2,126	2.375	4.90 **	0.068	0.481
Group × time × SOT condition	4,126	1.438	0.994	0.73	1.324

(**A**). MANOVA results for Jerk and RMS of COM. (**B**). ANOVA results for Jerk of COM and RMS of COM during single- and dual-task conditions. COM, center of mass; RMS; root mean square; ST, single-task; DT, dual-task; SOT, Sensory Organization Test. * *p* ≤ 0.05 ** *p* ≤ 0.01 *** *p* ≤ 0.001.

**Table 4:** Jerk and RMS of COM results under single- and dual-task conditions for each Sensory Organization Test condition.

**A**							
**Main Effects and Interaction**		**Single-Task Condition**					
	**Df (df1,df2)**	**Jerk of COM** ***F* Values**			**RMS of COM** ***F* Values**		
		**EOSS**	**ECSS**	**EOSSV**	**EOSS**	**ECSS**	**EOSSV**
Group effect	2,42	9.79 ***	15.11 ***	15.49 ***	4.35 *	1.179	3.184
Time effect	1,42	5.51 *	21.91 ***	12.255 ***	4.053	12.15 ***	15.97 ***
Group × time	2,42	0.81	6.42 **	5.56 **	4.98 *	4.61 *	3.90 *
**B**							
**Main Effects and Interaction**		**Dual-Task Condition**					
	**Df (df1,df2)**	**Jerk of COM** ***F* Values**			**RMS of COM** ***F* Values**		
Group effect	2,42	11.13 ***	31.22 ***	26.91 ***	9.98 ***	6.85 **	3.44 ***
Time effect	1,42	27.41 ***	11.56 ***	30.58 ***	11.77 ***	6.30 *	4.34 *
Group × time	2,42	4.94 *	7.05 **	13.43 ***	1.71	3.98 *	2.37

(**A**). ANOVA results for Jerk and RMS under single-task conditions during each Sensory Organization Test condition. (**B**). ANOVA results for Jerk and RMS under dual-task conditions during each SOT condition. COM, center of mass; RMS, root mean square; EOSS, eyes open sway referenced surface; ECSS, eyes closed sway references surface; EOSSV, eyes open sway refenced surface and vision. * *p* ≤ 0.05 ** *p* ≤ 0.01 *** *p* ≤ 0.001.

**Table 5:** Performance in cognitive interference and cognitive fatigue tasks.

	Older Adults Group		Stroke Group		Control	
	Baseline	Post-Intervention Task	Baseline	Post-Intervention Task	Baseline	Post-Intervention Task
CITP	24.01 ± 7.6	14.06 ± 6.4 **	21.2 ± 5.6	14.4 ± 6.8 **	20.6 ± 6.4	17.8 ± 5.01
CFTP (%of correct answers)	60.94		59.64		-	
Block 1	65.3 ± 17.3		68.5 ± 12.7		-	
Block 2	61.6 ± 27.3		61.6 ± 22.5		-	
Block 3	67.1 ± 23.4		50.1 ± 43.3		-	
Block 4	51.6 ± 39.9		63.7 ± 37.2		-	
Block 5	59.1 ± 28.6		54.3 ± 33.9		-	

Cognitive interference task performance and cognitive fatigue task performance of older adult group, stroke group, and control group. The cognitive interference task performance was calculated by subtracting the number of errors from the total number of subtractions. CITP, cognitive interference task performance; CFTP; cognitive fatigue task performance. Data are presented as the mean ± standard deviation. ** *p* ≤ 0.01.
